# Peritoneal Inflammation in PD-Related Peritonitis Induces Systemic Eryptosis: In Vitro and In Vivo Assessments

**DOI:** 10.3390/ijms25084284

**Published:** 2024-04-12

**Authors:** Grazia Maria Virzì, Niccolò Morisi, Davide Marturano, Sabrina Milan Manani, Ilaria Tantillo, Claudio Ronco, Monica Zanella

**Affiliations:** 1 Department of Nephrology, Dialysis and Transplant, St Bortolo Hospital, 36100 Vicenza, Italy; davide.marturano@aulss8.veneto.it (D.M.); sabrina.milan@aulss8.veneto.it (S.M.M.); ilaria.tantillo@aulss8.veneto.it (I.T.); monica.zanella@aulss8.veneto.it (M.Z.); 2 IRRIV—International Renal Research Institute, 36100 Vicenza, Italy; morisin@unimore.it (N.M.); cronco@goldnet.it (C.R.); 3 Nephrology Dialysis and Renal Transplantation Unit, University of Modena and Reggio Emilia, 41121 Modena, Italy

**Keywords:** eryptosis, peritoneal dialysis, peritonitis, cytokines, inflammation, induction of eryptosis

## Abstract

Erythrocytes (RBCs) have a highly specialized and organized membrane structure and undergo programmed cell death, known as eryptosis. Our preliminary data show a significant increase in the eryptosis during peritoneal dialysis (PD)-associated peritonitis. The objectives of the present study were assessment of the incrementation of eryptosis in PD patients with peritonitis, evaluation of the relationship between systemic eryptosis in peritonitis and specific peritonitis biomarkers in PD effluent (PDE), and confirmation of the induction of eryptosis by peritonitis in a vitro setting. We enrolled 22 PD patients with peritonitis and 17 healthy subjects (control group, CTR). For the in vivo study, eryptosis was measured in freshly isolated RBCs. For the in vitro study, healthy RBCs were exposed to the plasma of 22 PD patients with peritonitis and the plasma of the CTR group for 2, 4, and 24 h. Eryptosis was evaluated by flow cytometric analyses in vivo and in vitro. PDE samples were collected for biomarkers analysis.The percentage of eryptotic RBCs was significantly higher in PD patients with peritonitis than in CTR (PD patients with peritonitis: 7.7; IQR 4.3–14.2, versus CTR: 0.8; IQR 0.7–1.3; *p* < 0.001). We confirmed these in vivo results by in vitro experiments: healthy RBCs incubated with plasma from PD patients with peritonitis demonstrated a significant increase in eryptosis compared to healthy RBCs exposed to plasma from the control group at all times. Furthermore, significant positive correlations were observed between eryptosis level and all analyzed peritoneal biomarkers of peritonitis. We investigated a potential connection between systemic eryptosis and peritoneal biomarkers of peritonitis. Up-regulation of inflammatory markers could explain the increased rate of systemic eryptosis during PD-related peritonitis.

## 1. Introduction

Peritoneal dialysis (PD)-related peritonitis is a common complication of PD, often leading to a significant morbidity, catheter loss, transfer to hemodialysis, transient loss of ultrafiltration, possible permanent membrane damage, and occasionally death, especially in the first two years of treatment. Peritonitis may be directly related to peritoneal dialysis or secondary to a non-dialysis-related intra-abdominal or systemic process among peritoneal dialysis patients. Most cases are peritoneal dialysis-related [1,2,3]. In particular, peritonitis is responsible for about 20% of the failure of the PD [4]. Furthermore, numerous studies have highlighted the important correlations between peritonitis and increased mortality risk, reporting the six-fold rise in peritonitis in the month before the death [5,6,7]. Moreover, PD per se is accountable for chronic inflammation, especially for the composition and the non-absolute biocompatibility of dialysis solutions. In particular, patients on maintenance PD have increased intra-peritoneal levels of cytokines, such as interleukin (IL)-1β, IL-6, and transforming growth factor-β (TGF-β) [8]. Within the context of chronic systemic inflammation, infectious episodes such as bacterial peritonitis further exacerbate the inflammatory state [8]. Inflammation is the natural defense of our body involving cascades of immediate immunological responses and specific molecules (i.e., cytokines, chemokines, etc.) towards many harmful stimuli, including pathogens, necrotic cells, injury, or irritants. Acute inflammation is a protective machinery by which the injurious stimuli will be removed, and the healing process initiated. On the other hand, chronic inflammation develops if the conditions causing acute inflammation are not resolved over a period of time. Interestingly, chronic inflammation may be due to unnecessary physiological responses, such as the wound repairing process, which are intrinsically essential for maintaining a normal life.

Red blood cells (RBCs) are specialized organelle-free cells with a unique asymmetric membrane structure and are packaged with iron-containing hemoglobin, for the transport of oxygen [9]. Despite lacking a nucleus and mitochondria, RBCs can undergo premature stress-induced RBC death called eryptosis (similar to apoptosis). Eryptotic mechanism is necessarily activated to remove defective, damaged, or infective erythrocytes from the circulation [10]. This mechanism is different from cellular senescence (RBC life span is approximately 120 days) and/or accidental hemolysis (=the rupturing/lysis of erythrocytes and the release of their contents into surrounding fluid and/or bloodstream [11]. Although lacking the central apoptotic machinery, eryptosis shares characteristics like apoptosis of nucleated cells, such as cellular shrinkage, cell membrane blebbing, and scrambling with exposure of phosphatidylserine (PS) on the cell surface [12,13].

Multiple conditions can trigger eryptosis, such as increased cytosolic Ca (2+) concentration, iron deficiency, diabetes, kidney disease, sepsis, and infection of plasmodium [10,14,15,16]. In addition, eryptosis is related to the presence of toxic substances (i.e., uremic toxins such as vanadate, methylglyoxal, acrolein, indoxyl sulfate, urea, p-cresol) or the unbalance of redox state and inflammation mediators (cytokines such as IL-1β and IL-6) [12,17,18]. Uremia, inflammation, and hypoxia can induce PS exposure by flip-flop mechanism, bringing an increased rate of eryptosis, as proved in chronic hemodialysis (HD) patients with prolonged intradialytic hypoxemia [19]. A high level of eryptosis is also reported in chronic kidney disease (CKD), HD, and PD patients: in fact, eryptosis is triggered in part by a dialyzable plasma component and by dialysis procedure [20,21,22]. Recently, our group analyzed eryptosis in a PD stable population (46 patients and 17 controls) and reported an increase in eryptosis levels associated with the progressive residual diuresis and residual glomerular filtration rate (rGFR) loss, probably due to decreased uremic toxins clearance [22]. In addition, eryptosis levels were not affected by diabetes, hypertension, or cardiovascular disease in these PD [22].In addition, our group evaluated eryptosis levels in PD patients with and without peritonitis (study population: 65 PD patients: 34 PD patients without systemic inflammation nor PD-related peritonitis in the previous 3 months, and 31 PD patients with an acute episode of PD-related peritonitis)and their relationship with systemic conventional(C-reactive protein) and unconventional indices (cytokines) [23]. In particular, our preliminary data show that there is a significant increase in eryptosis levels in PD patients with peritonitis and significant positive correlations between the percentage of eryptosis and all systemic inflammatory markers tested (C-reactive protein, IL-1β and IL-6) [23]. Based on these data, we hypothesized a role for the classical and non-classical inflammatory mediators as promoters/inducers of the RBCs death in PD-related peritonitis.

Based on our previous observations and results about eryptosis and systemic inflammatory markers in PD stable patients and in PD patients with peritonitis, the objectives of the present study were assessment and confirmation of the incrementation of eryptosis in PD patients with peritonitis, evaluation of the relationship between systemic eryptosis in peritonitis and specific peritonitis biomarkers in PD effluent (PDE), and verify the induction of eryptosis by peritonitis in a vitro setting (in vitro models are usually employed to investigate cell or tissue senescence and apoptosis).

## 2. Results

### 2.1. Subjects Baseline Characteristics

A cohort of 22 chronic PD patients with peritonitis and 17 healthy controls were included in this observational project. A total of 10/22 PD patients were females. Regarding PD treatment, 13/22 PD patients were treated with continuous ambulatory PD (CAPD) and nine with automated PD (APD). The median vintage of PD treatment was 28.1 months (IQR 17.9–40.9) ranging from a minimum of 3 to a maximum of 124.9 months. In the PD population, end-stage renal disease (ESRD) was attributed to diabetic nephropathy (six patients), hypertension (four patients), glomerulosclerosis (three patients), nephroangiosclerosis (four patients), other causes (four patients) or unknown causes (one patient). Among PD patients, hypertension was the most common comorbidity found (eighteen patients), followed by diabetes (nine patients) and cardiovascular diseases (CVD) (nine patients). The median daily urine volume was 550 mL (375–1300) in the entire PD population. In particular, 10/22 patients had a residual diuresis greater than 500 mL.A total of 8/20 patients had Kt/V value < 1.7. Unfortunately, the Kt/V value was not available for two patients. A total of 15 PD patients had tClCr ≥ 45 L/wk/1.73 m^2^. No patients died and no patients received kidney transplantation during the study period.

The clinical, laboratory, and dialysis-related parameters of all 22 PD patients are briefly reported in Table 1.

For peritonitis resolution, all patients were promptly treated with intraperitoneal broad-spectrum antibiotics, according to current guidelines. PD patients clinically recovered from peritonitis after a mean of 13.2 ± 4.3 days. A total of 18/22 of the subjects had a first episode of peritonitis and responded to first-line antibiotics, whereas 4/22 had a relapsing episode of peritonitis, but subsequently responded to a second course of intraperitoneal antibiotics. Unfortunately, five subjects required catheter removal because of refractory peritonitis and transfer to HD. A total of 21/22 patients were treated at home, otherwise, one patient was admitted to the hospital for social reasons.

### 2.2. In Vivo Eryptosis Evaluation in PD Patients with Peritonitis

**Figure 1:**
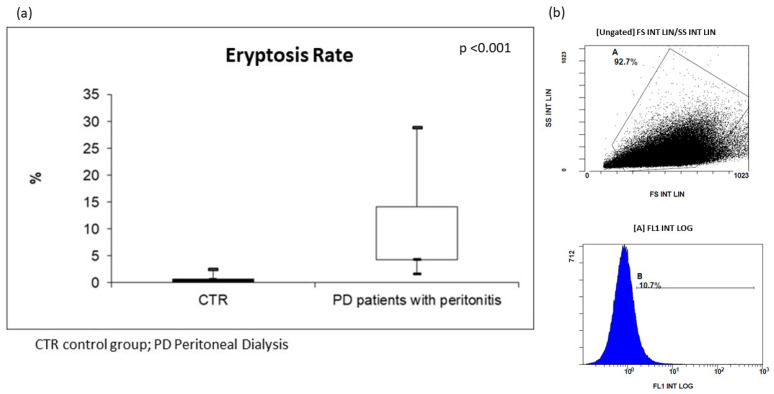
(**a**) Levels of eryptosis in PD patients with peritonitis and healthy controls; (**b**) two graphs extrapolated by flow cytometric analysis of PD patient with peritonitis.

For the in vivo assessment of eryptosis, we evaluated cellular modification (such as cell shrinkage, and cell membrane scrambling) and PS exposure at the RBC surface. Cell volume was determined utilizing flow cytometry; in particular, forward scatter (FSC) was taken as a measure of this parameter, reflecting cell volume and surface. Side scatter (SSC) measurement was used to have information about the internal complexity (i.e., granularity) of a cell. Based on our analysis, the RBCs of all PD subjects resulted dramatically modified in their cellular morphology. The median value of FSC was significantly higher in erythrocytes from these patients than in CTR. In order to analyze PS exposure at the RBC surface and quantify eryptotic RBCs, eryptotic RBCs were identified using FITC-AnnexinV-binding in flow cytometric analyses. As shown in Figure 1, the percentage of AnnexinV-binding RBCs was significantly higher in PD patients with peritonitis than in CTR (PD patients with peritonitis: 7.7%; IQR 4.3–14.2, versus CTR: 0.8%; IQR 0.7–1.3; *p* < 0.001) (Figure 1).

The median percentage of eryptosis did not differ significantly between males and females (*p* = 0.83), patients with and without diabetes (*p* = 0.44), with hypertension and without (*p* = 0.93), with CVD and without (*p* = 0.73). In addition, the median percentage of eryptosis showed no significant differences between patients treated with CAPD/APD (*p* = 0.15), with Kt/Vurea value ≤ 1.7 (n = 8/20) and > 1.7 (*p* = 0.31). Furthermore, there was no significant difference in the value of eryptosis on the first day of peritonitis in patients with (n = 4) or without a relapsing episode of peritonitis (*p* = 0.32). Identically, eryptosis did not differ in subjects with (n = 5) or without a refractory episode of peritonitis (*p* = 0.21).

### 2.3. Peritoneal Biomarkers in PD Patients with Peritonitis and Correlations with Eryptosis

WBC, NGAL, and cytokines levels in PDE are reported in Table 2. We observed significant positive correlations between pWBC and cytokines (IL-1β: Spearman’s rho = 0.31, *p* = 0.05; IL-6: Spearman’s rho = 0.3, *p* = 0.05). Furthermore, we reported positive strong correlations between pNGAL and cytokines levels (IL-1β: Spearman’s rho = 0.64, *p* = 0.001; IL-6: Spearman’s rho = 0.60, *p* = 0.005).

**Figure 2:**
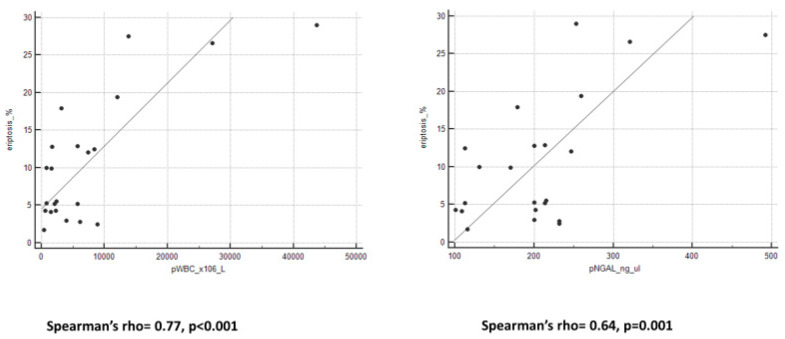
Graphs of correlation between eryptosis and pWBC and pNGAL.

**Figure 3:**
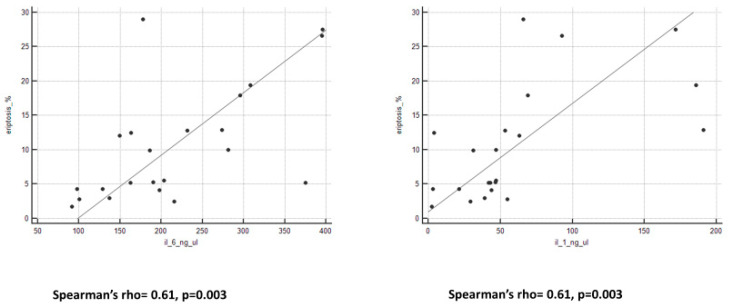
Graphs of correlation between eryptosis and cytokines.

We observed an important and interesting correlation between eryptosis levels and all peritoneal biomarkers (Figure 2 and Figure 3). Spearman’s rho and *p*-value are reported in Table 3.

### 2.4. In Vitro Exposure to Peritonitis Plasma and Induction of Eryptosis

**Figure 4:**
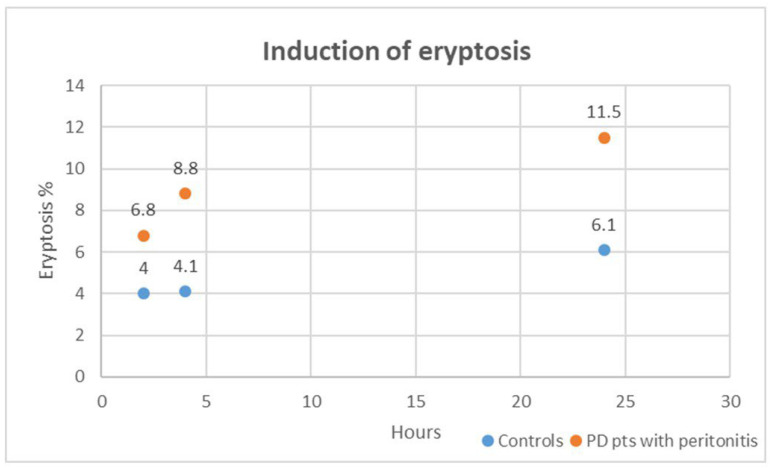
Induced eryptosis in our in vitro experiment. The case groups are represented in orange; the control groups are represented in blue.

In our in vitro exploration, we delved into the cytotoxic impact of plasma obtained from PD patients with peritonitis on healthy RBCs over varying exposure durations of 2, 4, and 24 h. Strikingly, when compared to healthy RBCs treated with plasma from the control group (healthy subjects without CKD and other comorbidities), those incubated with plasma from PD patients with peritonitis exhibited a significant distortion in cell morphology and a substantial surge in eryptosis at all time points (all, *p* < 0.001). Figure 4 shows induced eryptosis in our in vitro experiment. The cytotoxic effects unleashed by plasma from PD patients with peritonitis outperformed those induced by the control group at each time interval (all, *p* < 0.01). We specifically analyzed cytotoxic effects induced by plasma from PD patients with peritonitis. The eryptotic effect resulted significantly different: at 2 h compared to 4 h (*p* = 0.003), at 4 h compared to 24 h (*p* = 0.02), and at 2 h compared to 24 h (*p* = 0.001). For these cases, in our in vitro setting, the percentage of eryptosis resulted higher after the longer exposition (24 h).

## 3. Discussion

In this observational case-control study, we explored eryptosis in PD-related peritonitis employing both in vivo and in vitro methodologies. Flow cytometric investigations were used in order to detect and quantify eryptosis. Our multiple approaches sought to unravel the intricacies of eryptosis by first investigating its correlation with specific peritonitis biomarkers in peritoneal dialysis effluent (PDE), including peritoneal white blood cell count (pWBC), pNGAL and peritoneal cytokines, on the first day of peritonitis in PD patients. Furthermore, we explored the in vitro induction of eryptosis by exposing healthy red blood cells (RBCs) to plasma from PD patients with peritonitis at different time intervals, while closely monitoring the resulting cytotoxic effects. We choose the use of in vitro models because, in literature, this approach is usually employed to investigate the induction of cell apoptosis and senescence in different cell types. In particular, some studies reported the utility of in vitro evaluations to evaluate cytotoxic effects and eryptosis on RBC [12,16,17,18,19].

The eryptotic process is the premature, stress-induced death of damaged RBCs, comparable to apoptosis of nucleated cells, which is distinguished from accidental hemolysis or cellular senescence. Cell shrinkage, cell membrane scrambling, vesiculation, and PS translocation at the RBC surface are typical features characterizing eryptotic mechanisms. For these argumentations, eryptotic RBCs were identified and measured by cell volume dimension and AnnexinV-binding PS thought-out flow cytometric method. Eryptosis is a systemic condition affecting various different clinical conditions, such as anemia, diabetes, uremia, sepsis, fever, and dehydration [10,24]. Furthermore, recent research and several studies demonstrated strong correlations between this process and inflammatory state and/or oxidative stress condition [25,26,27].

For our in vivo study, we enrolled 22 PD patients on the first day of diagnosis of acute peritonitis and 17 healthy subjects without CKD or other comorbidities, as control group. We evaluated in vivo eryptosis percentage in blood fresh samples and pWBC, pNGAL, and peritoneal cytokines (IL-1 β and IL-6), as specific peritoneal markers of peritonitis. Our in vivo results reported higher levels of eryptosis in PD patients with peritonitis and confirmed our previous data demonstrating enhanced eryptosis levels in PD patients with PD-related peritonitis compared with PD stable patients (without peritonitis and inflammatory events in the last 3 months) [23]. In our previous work, positive correlations between systemic conventional and unconventional inflammatory markers and eryptosis percentage were reported [18,23]. In addition, in this report, we observed positive strong correlations between eryptosis and peritoneal biomarkers to definitively connect systemic eryptosis with specific local inflammatory molecules in PD-related peritonitis. The in vitro experiment confirmed all of these findings: the induction of eryptosis is triggered by cytokines and inflammatory markers. Specifically, we demonstrated the cytotoxic effect of plasma from PD patients with peritonitis on healthy RBCs at various time points. In particular, a significantly higher cell death rate was highlighted for RBCs incubated for extended time and prolonged exposition with higher concentrations of molecules.

Our in vitro results confirmed that eryptosis is mostly influenced by blood composition. In the broader context of eryptosis induction, it is noteworthy that several studies have confirmed the role of circulating elements in the bloodstream as instigators of eryptosis. However, the identification of a single protagonist has proved elusive in the existing literature. Several factors, including uremic toxins, inflammatory mediators, and redox imbalances, have been suggested to contribute to the complex cascade leading to eryptosis. In this context, Voelkl J. and colleagues described improved eryptosis in healthy RBCs treated by exposure to plasma collected from end-stage kidney disease patients. In this report, authors hypothesized that this cytotoxic process was prompted by higher extracellular phosphate concentrations and by calcium-phosphate complex formation [28]. Unfortunately, we did not analyze this aspect in our population. Likewise, Abed M. et al. exposed in vitro erythrocytes to different concentrations of C-reactive protein and demonstrated that this molecule is a trigger of PS translocation and eryptosis [29,30]. In 2013, Ahmed M. S. discovered that exposure of healthy red blood cells to Acrolein, an uremic toxin, induces the phenomenon of eryptosis [31].

Kempe D.S. et al. induced in vitro eryptosis in erythrocytes from healthy volunteers after exposure to plasma from septic patients [32]. These results were recently corroborated by Marcello M. and colleagues [33] with similar observations. In addition, they reported a good relationship between levels of eryptosis in septic patients and Endotoxin Activity Assay (EAA), mortality, and other biological markers of inflammation and oxidative stress.

Our data showed significant important and interesting correlations between systemic eryptosis levels and all peritoneal biomarkers. Furthermore, based on our results in PD patients with peritonitis compared with PD patients without peritonitis, we observed elevated values of systemic C-reactive protein and elevated values of peritoneal inflammatory markers. We hypothesized that during peritonitis, the intraperitoneal inflammation, with increased levels of pWBCs, pNGAL, and cytokines, could be strictly connected to a higher eryptotic rate and a systemic effect on RBCs. The actors of this cytotoxic effect could be local peritoneal cytokines: previous studies demonstrated that IL-1β and IL-6 induce structural and morphological changes in RBCs, eryptosis, and hypercoagulability [18,25]. In conclusion, we performed an in vivo and an in vitro study to confirm and validate increased eryptosis levels in PD-associated peritonitis and to investigate a potential connection between systemic eryptosis and peritoneal biomarkers of peritonitis. Future thematics of our study could be the sample size incrementation for in vivo and in vitro models, the introduction of more specific controls (for example: CKD patients, HD patients, PD patients without peritonitis), the quantification of oxidative stress and uremic toxins to better understand the induction of cytotoxic effect on RBCs.

In particular, we speculated that the eryptosis enhancement is directly linked with peritonitis, and based on the results, we theorized that the peritoneal membrane injury could be related to eryptosis. This point is of particular interest in both diagnostic and therapeutic fields. Up-regulation of peritoneal and systemic inflammatory markers could explain the increased rate of systemic eryptosis during PD-related peritonitis. In particular, we corroborated this point with our observations about in vitro induction of eryptosis by plasma from PD patients on the first day of peritonitis. Notably, the degree of eryptosis at the first day of peritonitis does not exhibit a direct correlation with the patient’s prognosis. Unfortunately, we did not collect multiple blood samples to investigate eryptosis development and modulation during peritonitis course. Despite the significant alterations in eryptosis levels observed in our study and the existing body of literature, establishing a straightforward link between the extent of eryptosis and patient outcomes remains elusive. In this context, our results can be considered hypothesis-generating, and stimulate further explorations.

## 4. Materials and Methods

### 4.1. Subjects

This is an observational case-control study over a 4-month period carried out in the Peritoneal Dialysis Unit within San Bortolo Hospital in Vicenza, Italy.

All patients older than 18 years and regularly treated by PD for a minimum treatment duration of 90 days were consequently eligible for this study. All patients were provided with information about the experimental protocol and the study’s aims. No patient withdrew their written consent, and there were no exclusions from the enrolled patient pool since the initiation of the study protocol.

Finally, a total of 22 PD patients with acute peritonitis were enrolled on the first day of diagnosis. Clinical, biochemical, and PD-related characteristics were recorded for all PD patients.

PD-related peritonitis was defined according to the Guidelines of the International Society of Peritoneal Dialysis (ISPD) [34]. It was characterized by a cloudy effluent with at least 100 leukocytes/L, of which more than 50% were polymorph nuclear cells, generally with abdominal pain and/or a positive effluent culture [35]. The peritoneal white cell count (pWBC) was evaluated by an overnight collection of PDE in all patients. Relapsing peritonitis was defined as the development of peritonitis within 4 weeks of completion of antibiotic treatment for a prior episode with the same organism isolation or one sterile episode. Refractory peritonitis was defined as failure of the effluent to clear after 5 days of appropriate antibiotics, according to current guidelines [34,35]. For relapsing and refractory peritonitis, biological samples were collected from the patients at the first day of peritonitis presentation.

We additionally included 17 healthy volunteers without CKD and other comorbidities as control group (CTR) in this study. All patients were enrolled on the same day in the Blood Center of San Bortolo Hospital and plasma from these subjects was kindly offered by the blood bank of San Bortolo Hospital.

### 4.2. Samples Collection

Blood and PDE samples from PD patients were taken at the time of peritonitis diagnosis for eryptosis and biomarkers analysis. Blood samples were collected into ethylenediaminetetra-acetic acid (EDTA) -containing tubes and processed within 1 h after venipuncture (centrifugation for 7 min at 1600× *g*).

### 4.3. Laboratory Parameters

Blood urea nitrogen, serum creatinine (sCr), and C-reactive protein (CRP) were measured by standard laboratory techniques with an automatic analyzer (Dimension Vista, Siemens Healthcare, Tarrytown, NY, USA). At the time of diagnosis, PDE samples were collected for the evaluation of pWBC, pNGAL (Neutrophil Gelatinase-Associated Lipocalin), and cytokines (IL-6 and IL-1β). White cell count in PDE was quantified by automated hematology analyzers XN 9000 (SYSMEX, Kobe, Japan). pNGAL was measured by the standard BioPorto test (BioPorto Diagnostics, 2900 Hellerup, Denmark), using particle-enhanced turbidimetric immunoassay for quantitative determination. The assay covers a range of 50 to 3000 μg/L, with values equal to or exceeding 120 ng/mL considered indicative of peritonitis for our clinical practice [36,37].

Quantifications of cytokines (IL-1β and IL-6) in PDE were performed by ELISA (=Enzyme-Linked Immunosorbent Assay) kit according to the protocol’s indications. Quantitative determination of plasma IL-6 was executed by Human IL-6 Simple step ELISA Kit- ab178013 (Discovery Drive, Cambridge Biomedical Campus, Cambridge, CB2 0AX, UK). Quantitative determination of plasma IL-1β was executed by Human IL-1β Simple step ELISA Kit- ab214025 (Discovery Drive, Cambridge Biomedical Campus, Cambridge, CB2 0AX, UK). Optical density was read by VICTORX4 Multilabel Plate Reader (PerkinElmer Life Sciences, Waltham, MA, USA) at 450 nm for both cytokines. Concentrations of Il-1β and IL-6 were extrapolated by interpolation with the standard curve based on the manufacturer’s instructions. Standard samples ranged from 7.8 to 500 ng/mL for IL-1β and from 3.1 to 200 ng/mL for IL-6. Human IL-1β Instant ELISA Kit sensitivity is 0.7 ng/mL pg/mL. Human IL-6 Instant ELISA Kit sensitivity is 0.92 ng/mL. All tests were carried out in duplicate.

### 4.4. In Vivo Eryptosis

In vivo, eryptosis was evaluated in CTR and PD patients with peritonitis on the first day of peritonitis. A total of 1 μL of leukocyte-depleted RBCs was diluted in 400 μL Ringer Solution (=balanced polyionic nonalkalinizing isotonic crystalloid solution that contains physiologic concentrations of Na^+^, K^+^, Ca_2_^+^, and Cl^−^). A total of 1μL of AnnexinV-FITC-conjugated (Beckman Coulter, Brea, CA, USA) was used for eryptosis measurement (incubation for 20 mins in dark stained according to manufacturer’s instructions). Lastly, 400 μL of Ringer was added to each tube. 

### 4.5. In Vitro Exposure to Peritonitis Plasma and Induction of Eryptosis

We performed an in vitro study with healthy RBCs exposed to plasma of PD patients with peritonitis and CTR at 2, 4, and 24 h. Finally, we compared the induction of eryptosis rate after exposure of RBCs in these 2 groups at different time points.

For this in vitro experiment, 1.5 µL of healthy RBCs were plated in 48-well plates with 300 µL of RPMI-1640 supplemented with 2 mM L-glutamine, 100 IU/mL penicillin and 100 µg/mL streptomycin (all from Sigma Chemical Co., St. Louis, MO, USA). Each well was incubated by 10% EDTA-plasma from PD patients with peritonitis or CTR group. We used untreated RBCs as a negative control (supplemented RPMI-1640 and 10% of heat-inactivated Fetal Bovine (Serum-Sigma Chemical Co., St. Louis, MO, USA). All RBCs were incubated in standard conditions (37 °C in 5% CO^2^). Each incubation was performed for 2, 4, and 24 h in duplicate. Each well was tested twice. 

### 4.6. Flow Cytometry Evaluation

All eryptosis evaluations were made in fresh RBC samples. We detected forward scatter (FSC) and side scatter (SSC). FSC detects scatter along the path of the laser, and side scatter (SSC) measures scatter at a ninety-degree angle relative to the laser. FSC intensity is proportional to the diameter of the cell and is primarily due to light diffraction around the cell. FSC signal can be used for the discrimination of cells by size. SSC, on the other hand, is from the light refracted or reflected at the interface between the laser and intracellular structures, such as granules and nucleus. SSC provides information about the internal complexity (i.e., granularity) of a cell.

Furthermore, we used AnnexinV conjugated with FITC (=Fluorescein-5-isothiocyanate, excitation max. of 498 nm, emission max. of 517 nm). PS avidly binds AnnexinV, which is applied to mark and classify eryptotic cells [12,38]. PS exposure at the RBC surface was estimated from fluorescence detection (FITC-AnnexinV—Beckman Coulter, Brea, CA, USA) by Navios Flow Cytometer (Beckman Coulter, Brea, CA, USA). FITC-AnnexinV fluorescence intensity was measured with an excitation wavelength of 488 nm and an emission wavelength of 530 nm. RBCs were gated and counted by identifying those cells that exposed PS at the RBC surface. A minimum of 100,000 events were collected on each sample.

### 4.7. Statistical Analysis

Statistical analysis was performed using the SPSS Software package version 26.0 (IBM Corp., Armonk, NY, USA). A *p*-value of <0.05 was considered statistically significant. Categorical variables were expressed as percentages; continuous variables were expressed as mean ± standard deviation (parametric variables) or median and interquartile range (IQR) (non-parametric variables). For vintage of PD treatment, minimum and maximum were reported. Normality tests were performed by the Kolmogorov–Smirnov test.The Mann–Whitney U test or T test was used for comparison of two groups when appropriate. The Kruskal–Wallis test or ANOVA test for multiple comparisons was applied to compare the groups when appropriate. Spearman’s rho correlations were calculated to verify the correlation between variables.

## Figures and Tables

**Table 1:** Descriptive data for all 22 PD patients with peritonitis.

PD Patients with Peritonitis	
Age, years	63 ± 14
Sex	10/22 females
Height, cm	169 ± 8
CAPD	13/22
Months of PD	28.1, IQR 17.9–40.9
Diabetes	9/22
Hypertension	18/22
CVD	9/22
Daily urine Volume, ml	550, IQR 375–1300
Hb, g/L	117 ± 14
Albumin, g/dL	2.9 ± 0.4
Urea, mg/dL	116, IQR 90–144
CRP, mg/dL	5.5, IQR 2.0–9.5

**Table 2:** Biomarker levels in PDE.

Biomarkers in PDE	
pWBC, 106/L	1942.5, IQR 4823.5–8520.8
pNGAL, mg/mL	200, IQR 212–236
pIL-6, ng/mL	193.8, IQR 159.0–285.0
pIL-1β, ng/mL	46.8, IQR 37.1–66.8

**Table 3:** Correlations between eryptosis and biomarkers in PDE.

Correlations		
	Spearman’s Rho	*p*-Value
Eryptosis-pWBC	0.77	<0.001
Eryptosis-pNGAL	0.64	0.001
Eryptosis-pIL-6	0.61	0.003
Eryptosis-pIL-1β	0.64	0.001

## Data Availability

The data presented in this study are available on request from the corresponding author. The data are not publicly available due to our internal policy.
